# Supplementary material for: Diffusion-programmed catalysis in nanoporous material
Source: Nat Commun. 2025 Feb 3;16:1231. doi: 10.1038/s41467-025-56575-6 (PMC11790907; doi:10.1038/s41467-025-56575-6)
Supplement: Supplementary file 1 — Supplementary Information [file 41467_2025_56575_MOESM1_ESM.pdf]

Supporting Information

Diffusion-programmed catalysis in nanoporous material

Suvendu Panda,[1] Tanmoy Maity,[12] Susmita Sarkar,[1] Arun Kumar Manna,[1] Jagannath Mondal,[1] Ritesh Haldar[1*]

[1]Tata Institute of Fundamental Research Hyderabad, Gopanpally, Hyderabad 500046, Telangana, India

Present address: [2]Haldia Institute of Technology, Department of Applied Science and Humanities, Hatiberia, ICARE Complex, Haldia, Purba Medinipur, West Bengal, 721657, India

Email: riteshhaldar@tifrh.res.in

Supplementary methods

1. Experimental section

1.1. Chemicals and materials

Zirconium(IV) propoxide solution (70 wt% in 1-propanol, TCI), NH_2-bdc (TCI), , acetic acid (99.9%, SRL), *N*, *N'*-dimethylformamide (SRL), anhydrous methanol (Sigma Aldrich), DI water, benzaldehyde (Sigma Aldrich), ethyl cyanoacetate (TCI), *tert*-butyl cyanoacetate (TCI), ethanol (99.9%, Sigma) and 11-Mercapto-1-undecanol (Sigma), $ZrCl_4·8H_2O$ (TCI) were used without any further purification.

2.2. UiO-66-NH$_2$ MOF catalyst

The UiO-66-NH$_2$ MOF is constructed by linking a $Zr_6(\mu_3\text{-}O)_4(\mu_3\text{-}OH)_4$ node with 2-amino terephthalate linker. The node or secondary building unit (SBU), formed *in situ*, is kinetically and thermodynamically robust resulting in a highly stable framework structure. The cross-linking of the node and linker produces two distinct interconnected cages of dimensions 8 and 11 Å.[1] These cages are accessible through a window ~6 Å in dimension (Supplementary Fig. 1).

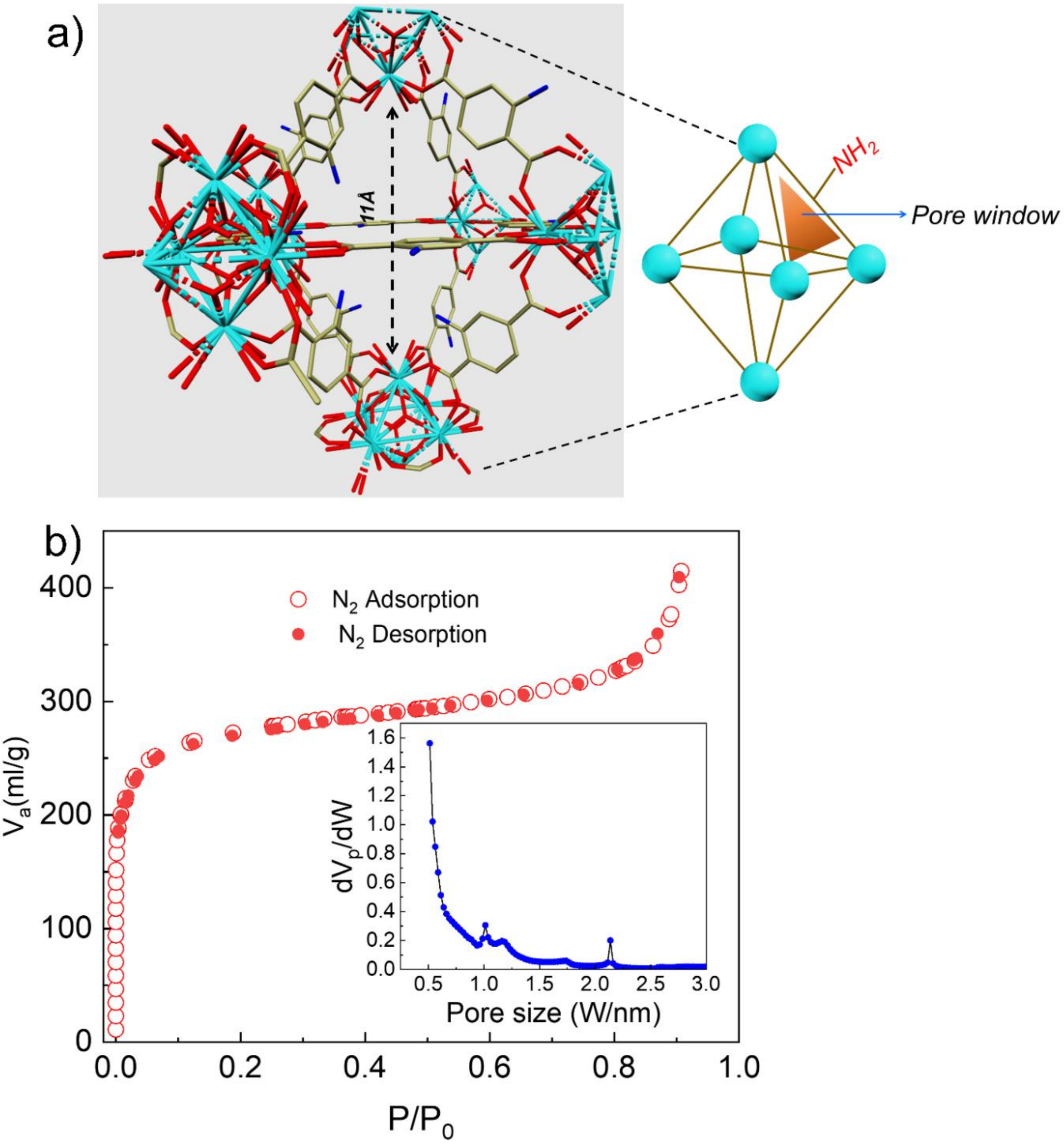

Supplementary Fig. 1. a) UiO-66-NH_2 structure with free -NH_2 functionality. b) N_2 adsorption isotherm of UiO-66-NH_2 powder. Synthesis conditions: 10.77 mM metal cluster solution (refer to Section 2.7 for details), 10.77 mM bdc-NH_2 solution in DMF, room temperature, 18 h.

2.3. Synthesis of $UiO-66-NH_2$ submicron-sized crystals using various modulator concentrations

$UiO-66-NH_2$ was synthesized according to literature procedures.[2] Different crystallite sizes of $UiO-66-NH_2$ were obtained by varying concentrations of the modulator.

Synthesis using 25 eqv modulator: In a glass beaker $ZrCl_4$ (233 mg, 1 mmol), acetic acid (1.5 mL, 25 mmol), and DMF (5 mL) were sonicated for 30 minutes and or until fully dissolved. Then 2-amino terephthalic acid (181 mg, 1 mmol) and DMF (5 mL) were added to the mixture and sonicated for an additional 30 min. After sonication, the entire solution was transferred to a 40 mL Teflon autoclave with a steel jacket and heated at 393 K. After 24 hours, the powder was separated by centrifugation and washed with DMF and ethanol. This was followed by a solvent exchange with ethanol for 24 hours. Subsequently, using a gradient separation process 160 ± 50 nm particles were isolated (Supplementary Fig. 2).

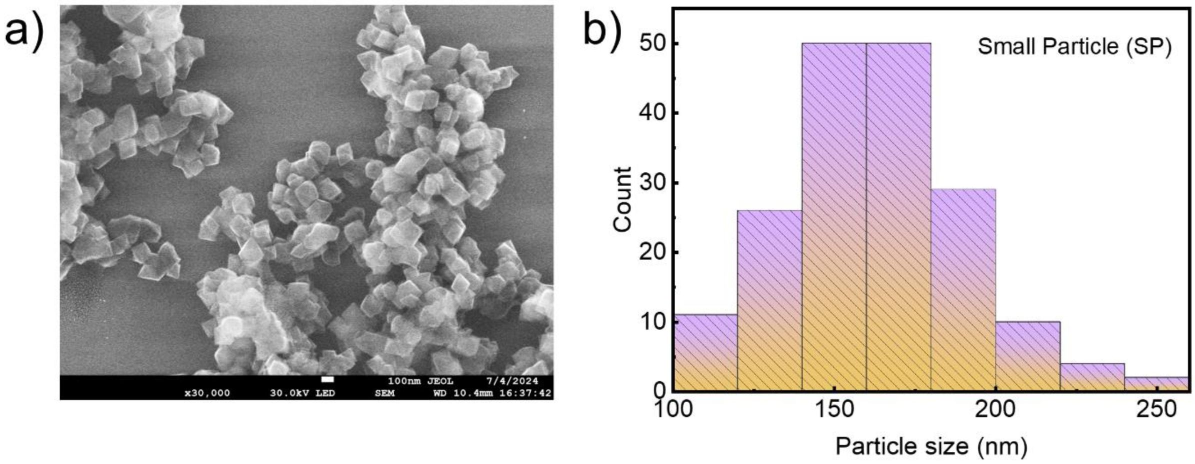

Supplementary Fig. 2. a) SEM morphology image (scale bar = 100 nm), b) crystallite size distribution of the $UiO-66-NH_2$.

Synthesis with 500 eqv modulator: In a glass beaker $ZrCl_4$ (233 mg, 1 mmol), acetic acid (30 mL, 500 mmol), and DMF (5 mL) were sonicated for 30 minutes and or until fully dissolved. Then 2-amino terephthalic acid (181 mg, 1 mmol) and DMF (5mL) were added to the mixture and sonicated for an additional 30 minutes. After sonication, the entire solution was transferred to a 40 mL Teflon autoclave with a steel jacket and heated at 393K. After 72 hours, the powder was separated by centrifugation and washed with DMF and ethanol. This was followed by a solvent exchange with ethanol for 24 hours. Subsequently, 500 ± 50 nm particles (Supplementary Fig. 3) were isolated by a gradient separation process.

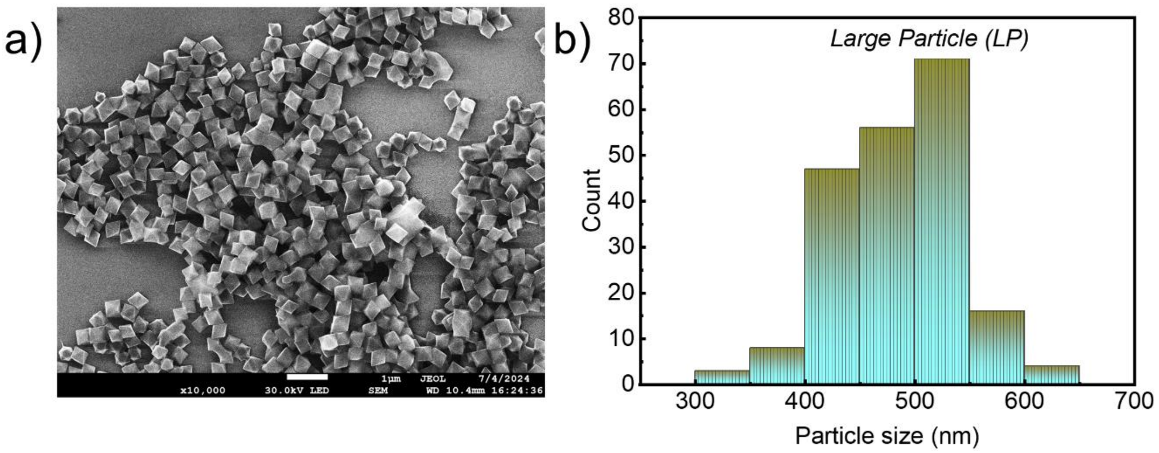

Supplementary Fig. 3. a) SEM morphology image (scale bar = 100 nm), b) crystallite size distribution of the UiO-66-NH_2.

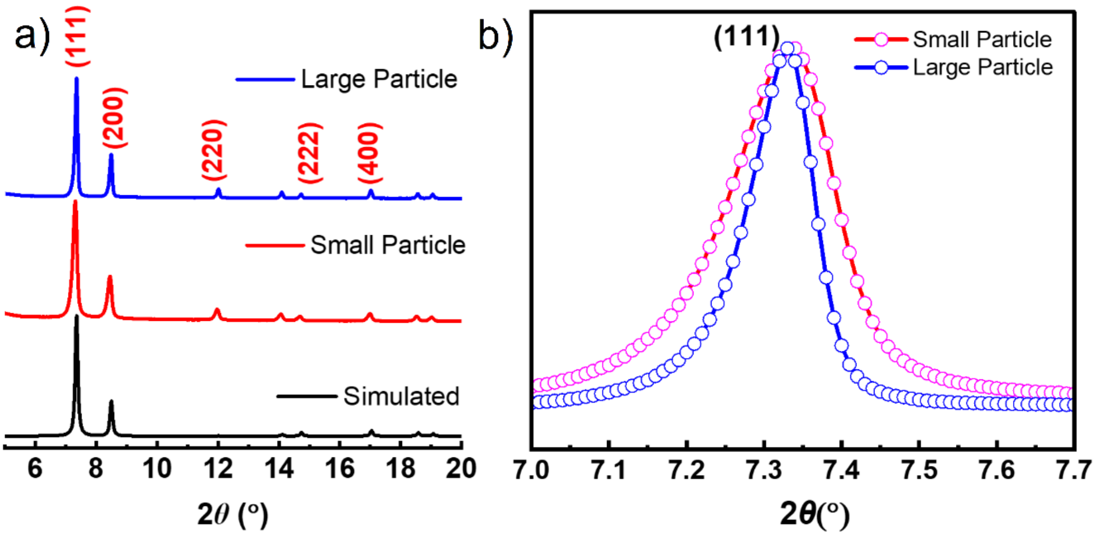

Supplementary Fig. 4. a) Comparison of XRD patterns of the UiO-66-NH_2 powder small particles (red) and large particles (blue) with the simulated XRD patterns. b) Zoomed XRD patterns depict full-width at half maxima (FWHM) is lower for large particles than small particles.

2.4. Synthesis of ethyl-2-cyano-3-phenylacrylate (*Et*-Acr)

In a 25 mL RB 1mmol of benzaldehyde, 1mmol of ethyl 2-cyanoacetate, and 9.2 μL of aniline were mixed in 10 mL of ethanol. The mixture was then stirred for 10 hours to obtain ethyl-2-cyano-3-phenylacrylate. Column chromatography was performed to isolate the pure

product. Yield 99%. ^{1}H-NMR (300 MHz, $CDCl_3$) δ ppm 8.26 (S, 1H), 7.96 (dd, J= 6 Hz, 2H), 7.5 (m, 3H), 1.58 (s, 9H).

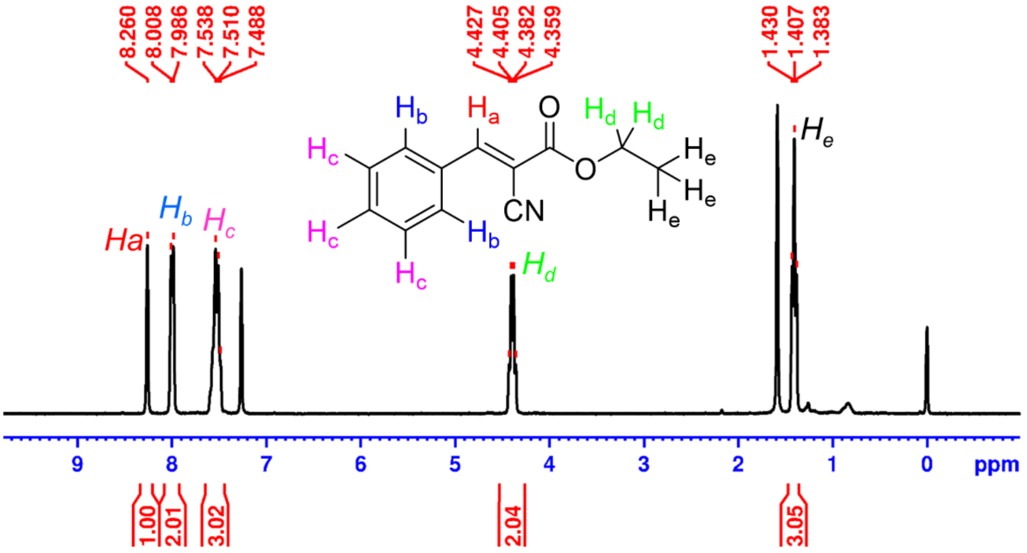

Supplementary Fig. 5. ^{1}H-NMR of ethyl-2-cyano-3-phenylacrylate compound.

2.5. Synthesis of *tert*-butyl-2-cyano-3-phenylacrylate (*t-but*-Acr)

In a 25mL RB 1mmol of benzaldehyde, 1mmol of *tert*-butyl-2-cyanoacetate, and 9.2µL of aniline were mixed in 10mL of ethanol. The mixture was then stirred for 30 hours to obtain *tert*-butyl-2-cyano-3-phenylacrylate. Column chromatography was performed to isolate the pure product. Yield 99%. ^{1}H-NMR (300MHz, $CDCl_3$) δ ppm 8.15 (S, 1H), 8 (dd, J= 6 Hz, 2H), 7.52 (m, 3H), 4.3 (q, J=6Hz, 2H), 1.4 (t, J = 6Hz, 3H).

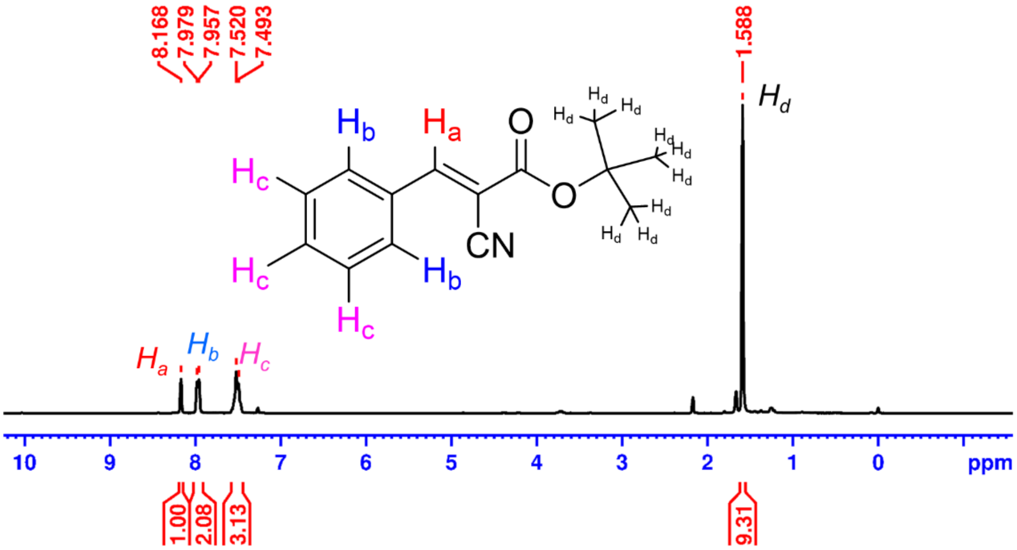

Supplementary Fig. 6. ^{1}H-NMR of pure *tert*-butyl-2-cyano-3-phenylacrylate compound.

2.6. Catalysis by using UiO-66-NH_2 powder

In a typical catalytic experiment, 21 mg (0.01mmol, 1% catalyst) of UiO-66-NH_2 (MW= 1754.1 g/mol; Formula: $Zr_6O_{30}C_{48}H_{24}N_6$) was placed in a flask and heated at 423K for 12 hours. After that, a solution of 1mmol benzaldehyde, 1mmol of active methylene compounds, and 10mL ethanol was added with stirring and heated to 343 K. After 24h, MOF nanoparticles were separated from the reaction mixture by using a syringe filter. The reaction yields, as shown in Table 1, were monitored using the NMR technique.

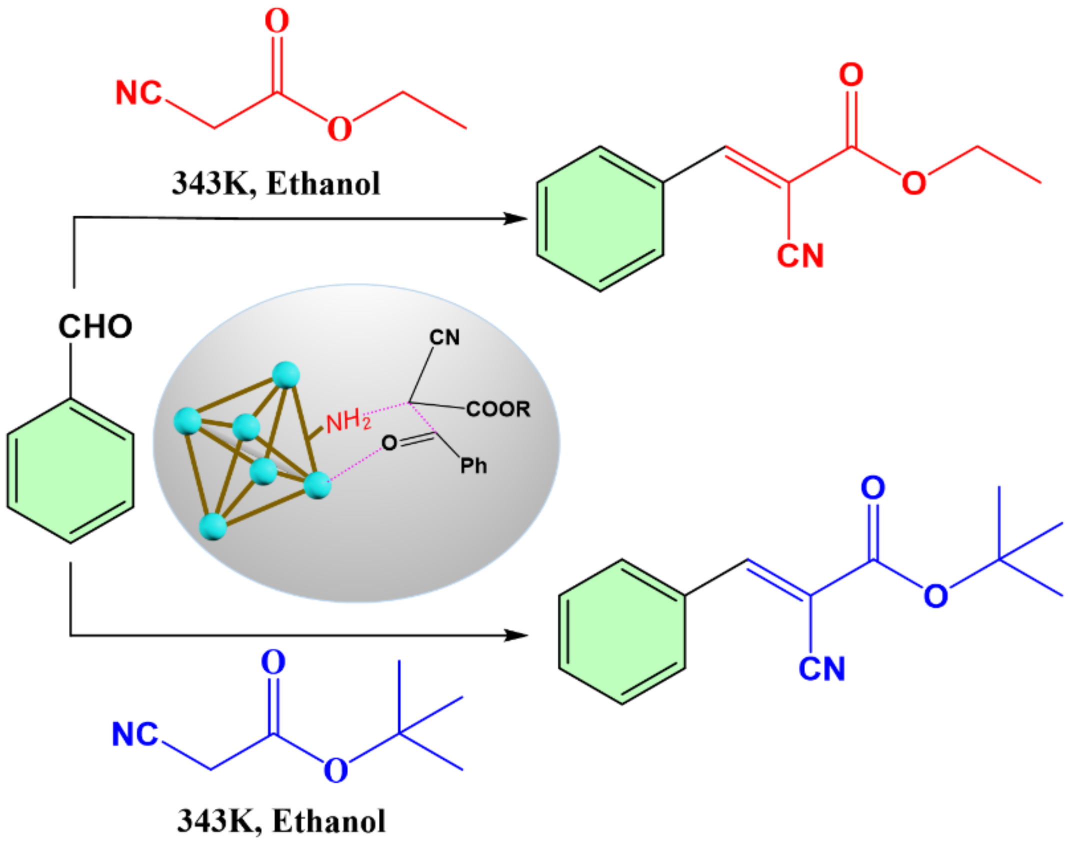

Supplementary Fig. 7. Plausible mechanistic pathway of the Knoevenagel condensation[3] using UiO-66-NH_2.

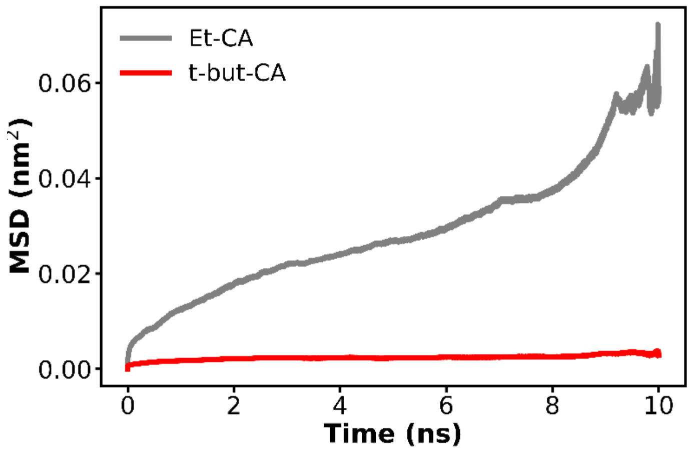

Supplementary Fig. 8. Time profile of mean square displacement (MSD) of *Et*-CA and *t-But*-CA through UiO-66-NH_2. Diffusion constants of *Et*-CA and *t-But*-CA are 0.0013515±0.0002945 nm^2/s and 0.00014345±0.0000403 nm^2/s respectively, which are averaged over multiple replicas

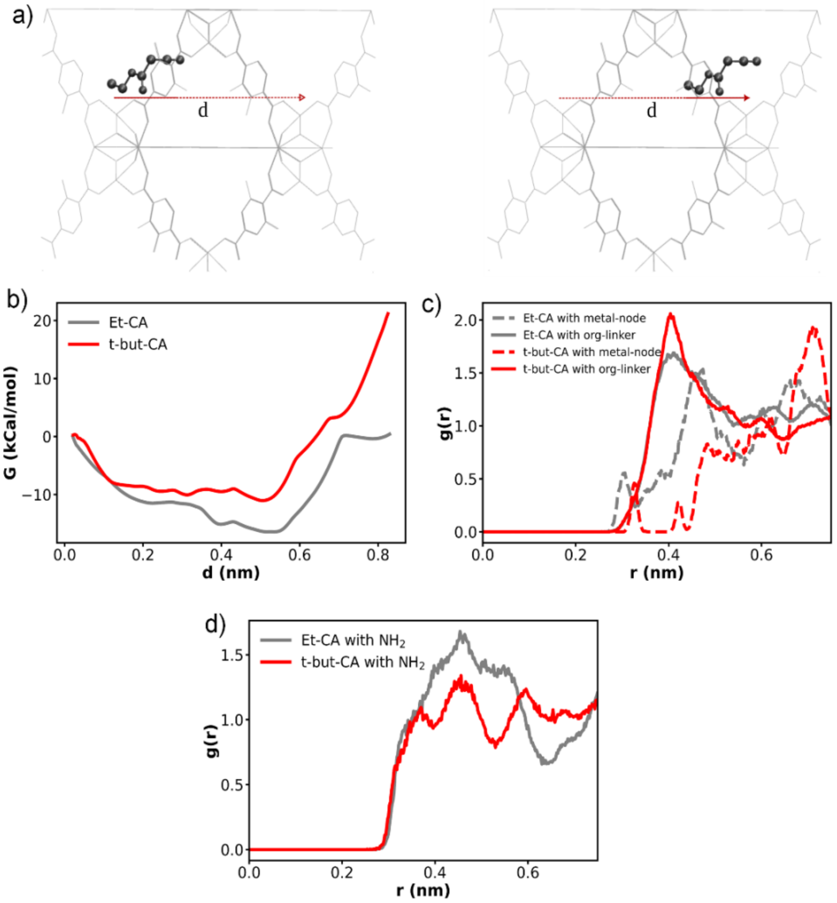

Supplementary Fig. 9. a) Figure shows the collective variable chosen for umbrella sampling simulation involving molecular movement from aperture of one pore to another for *Et*-CA

(represented in cpk representation). The same is considered for *t-But*-CA also; b) Free energy profile corresponding to molecular transport through a MOF pore compared for Et-CA and t-but-CA; c) Pair correlation functions of active -CH_2 group of *Et*-CA and *t-But*-CA (individually) with respect to metal-oxo nodes and bdc-NH_2 in solid and dashed lines respectively; d) Pair correlation function of active -CH_2 group of *Et*-CA and *t-But*-CA (individually) with respect to the -NH_2 group of organic linker in MOF.

Supplementary Table 1.**:** The impact of crystallite size on the UiO-66-NH_2 catalysed Knoevenagel condensation between benzaldehyde and an activated methylene compound.

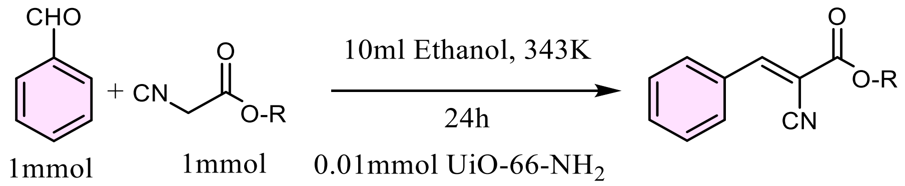

**Entry**	**Particle size of MOF**	**Active methylene compound**	**Yield (%)**	**TOF (h^{-1})**
**1**	160 ± 50 nm	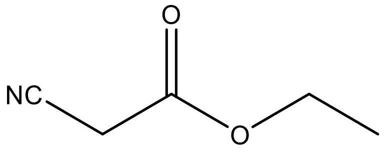	40	1.66
**2**	160 ± 50 nm	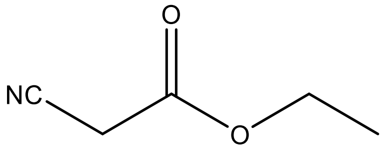	43.82	1.81
**3**	160 ± 50 nm	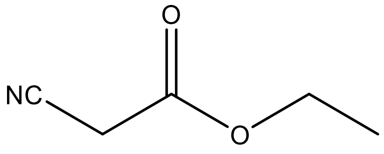	43.5	1.82
**4**	160 ± 50 nm	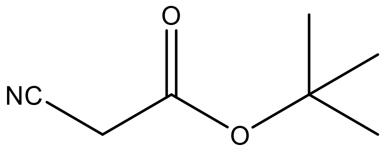	24	0.98

**5**	160 ± 50 nm	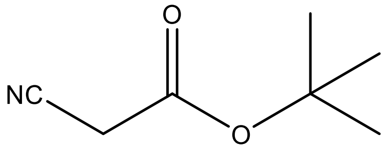	24	1
**6**	500 ± 50 nm	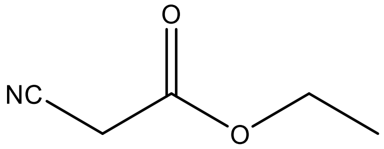	24	1
**7**	500 ± 50 nm	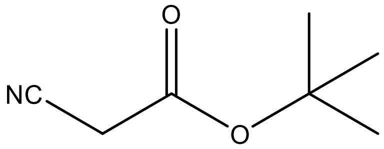	9	0.38

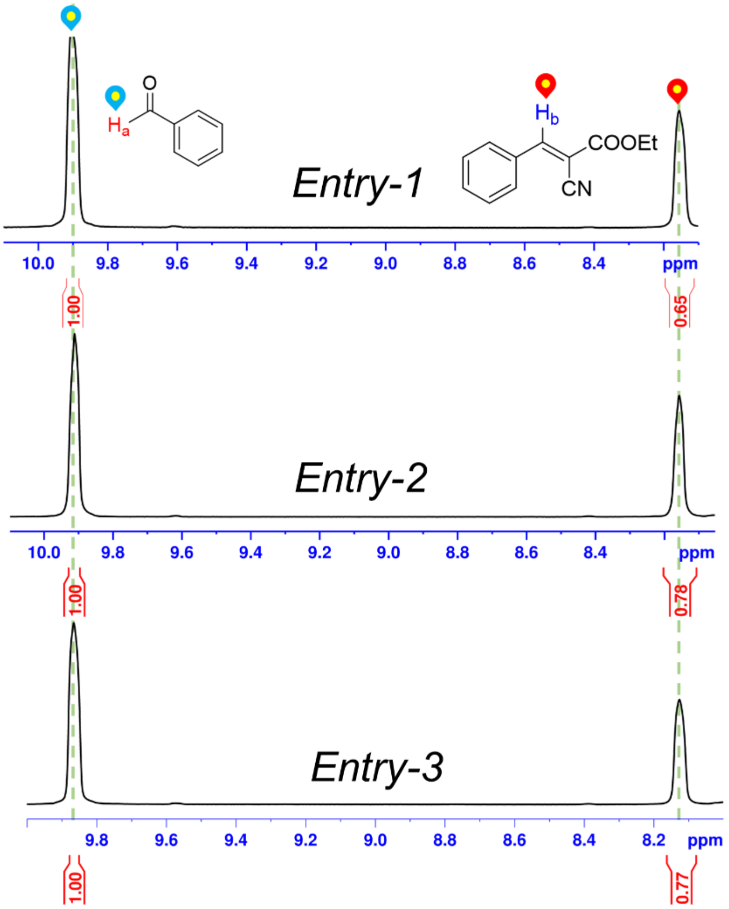

Supplementary Fig. 10. ^{1}H-NMR of Entry 1-3.

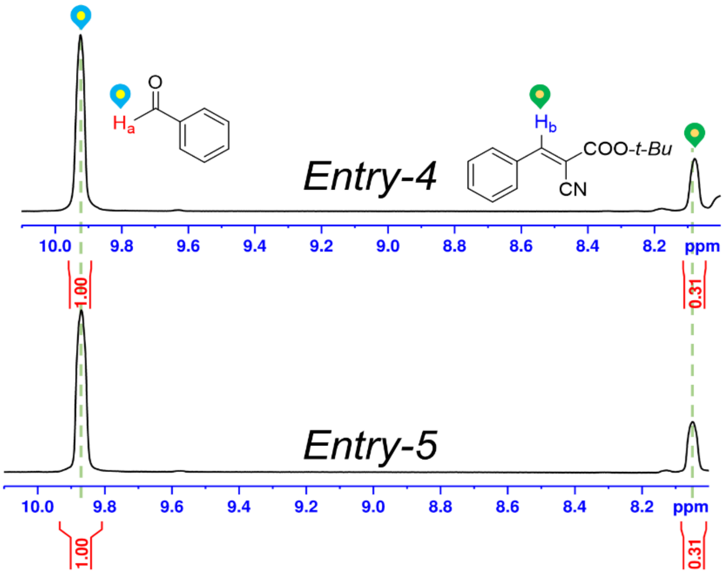

Supplementary Fig. 11. ^{1}H-NMR of Entry 4-5.

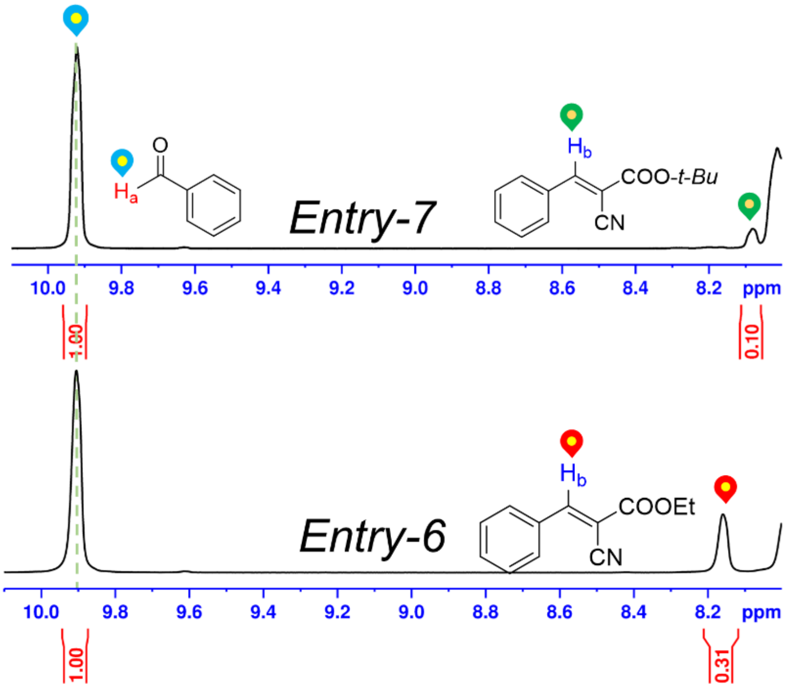

Supplementary Fig. 12. ^{1}H-NMR of Entry 6-7.

2.7. Synthesis of (Zr_6 (μ_3-O)$_4$(μ_3-OH)$_4$($OOCCH_3$)$_{12}$) metal cluster solution

Metal cluster solution was synthesized following an earlier reported method.[4] 71 µL of a 70% zirconium propoxide [Zr(O^{n}Pr)$_4$] solution in 1-propanol (0.0519 g, 0.158 mmol), 7 mL of DMF, and 4 mL of acetic acid (4.196 g, 70 mmol) mixture solution was heated in a 25 mL

round-bottom flask at 130 °C for 2 h. A noticeable change in solution from colorless to yellow was observed. Then the solution was allowed to cool down to room temperature (~ 298 K).

2.8. Synthesis of UiO-66-NH_2 thin films at room temperature

A precursor solution was prepared by mixing 100 µL of as-prepared metal cluster solution and 100 µL of 10.77 mM bdc-NH_2 solution (in DMF). A droplet (200 µL) of this precursor solution was mixed well and drop casted on a functionalized Si/SiO_2 (2 cm × 2 cm) substrate at ambient conditions (298 ± 3 K, 50-60% RH).[5] After 2.5-3 hours, the substrate was thoroughly washed and dried with N_2 flow to remove any excess deposition. The same procedure was repeated once more time to obtain UiO_{TF1}, twice to obtain UiO_{TF2}, and three times to obtain UiO_{TF3}.

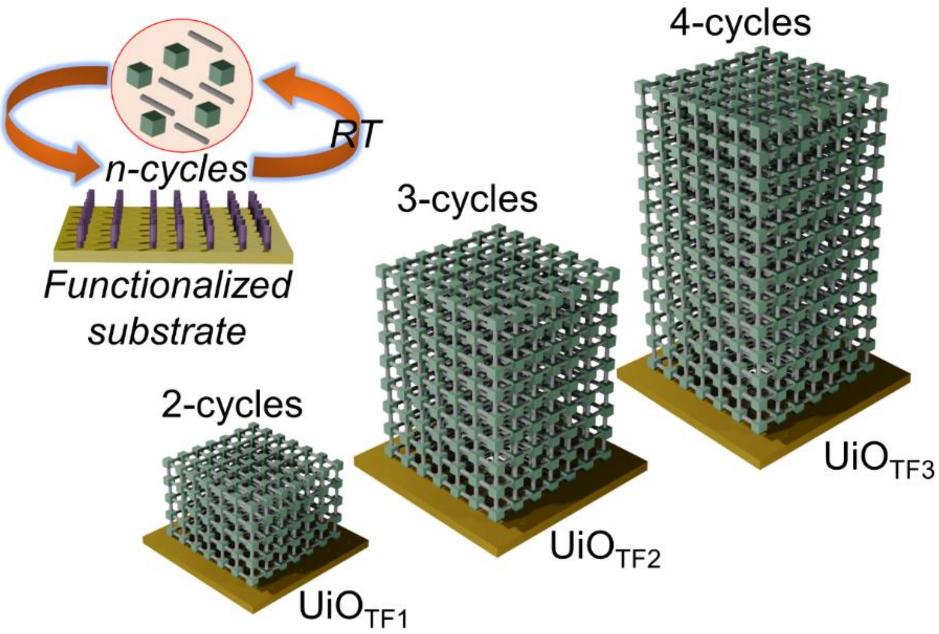

Supplementary Fig. 13. The synthesis scheme of UiO-66-NH_2 MOF thin film with controllable thickness. RT = room temperature.

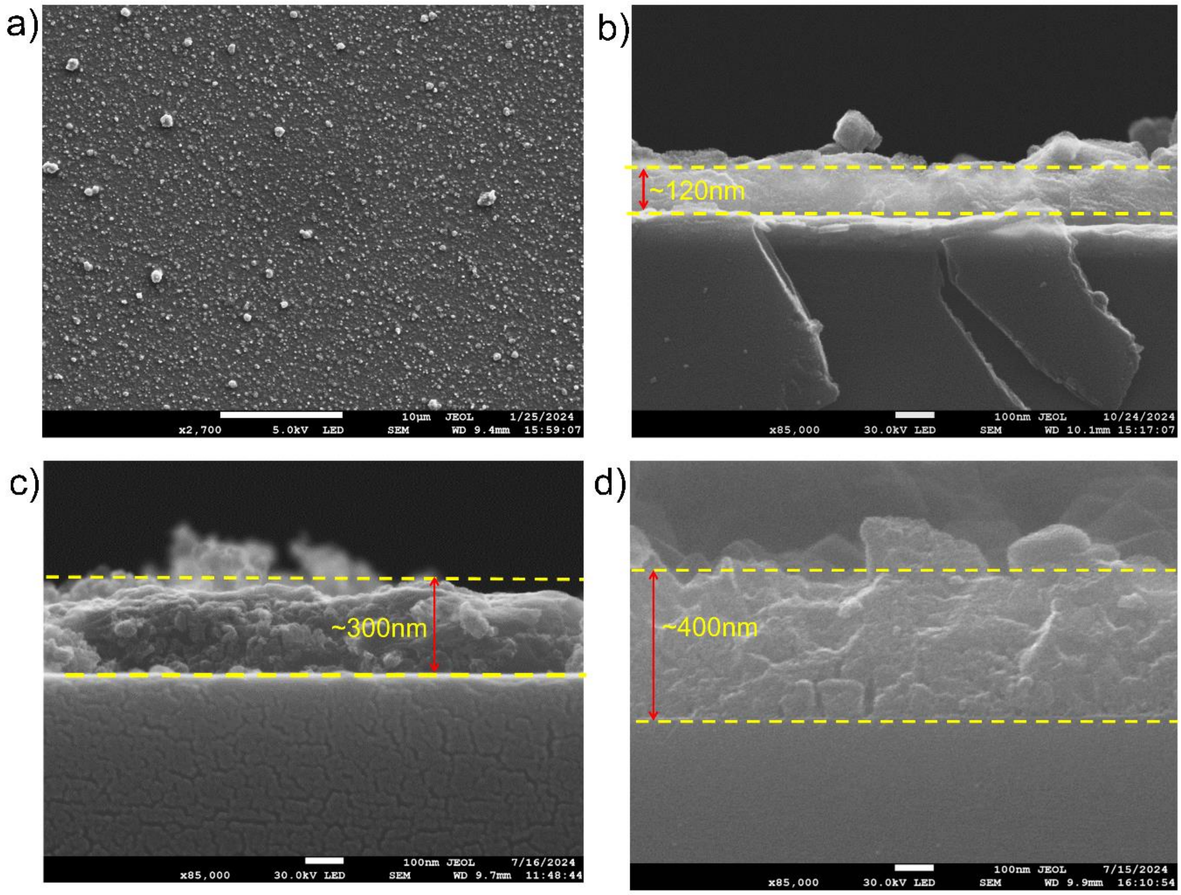

Supplementary Fig. 14. a) SEM morphology image of $UiO_{TF1.}$ SEM cross-section image of b) UiO_{TF1}, c) UiO_{TF2}, and d) UiO_{TF3}.

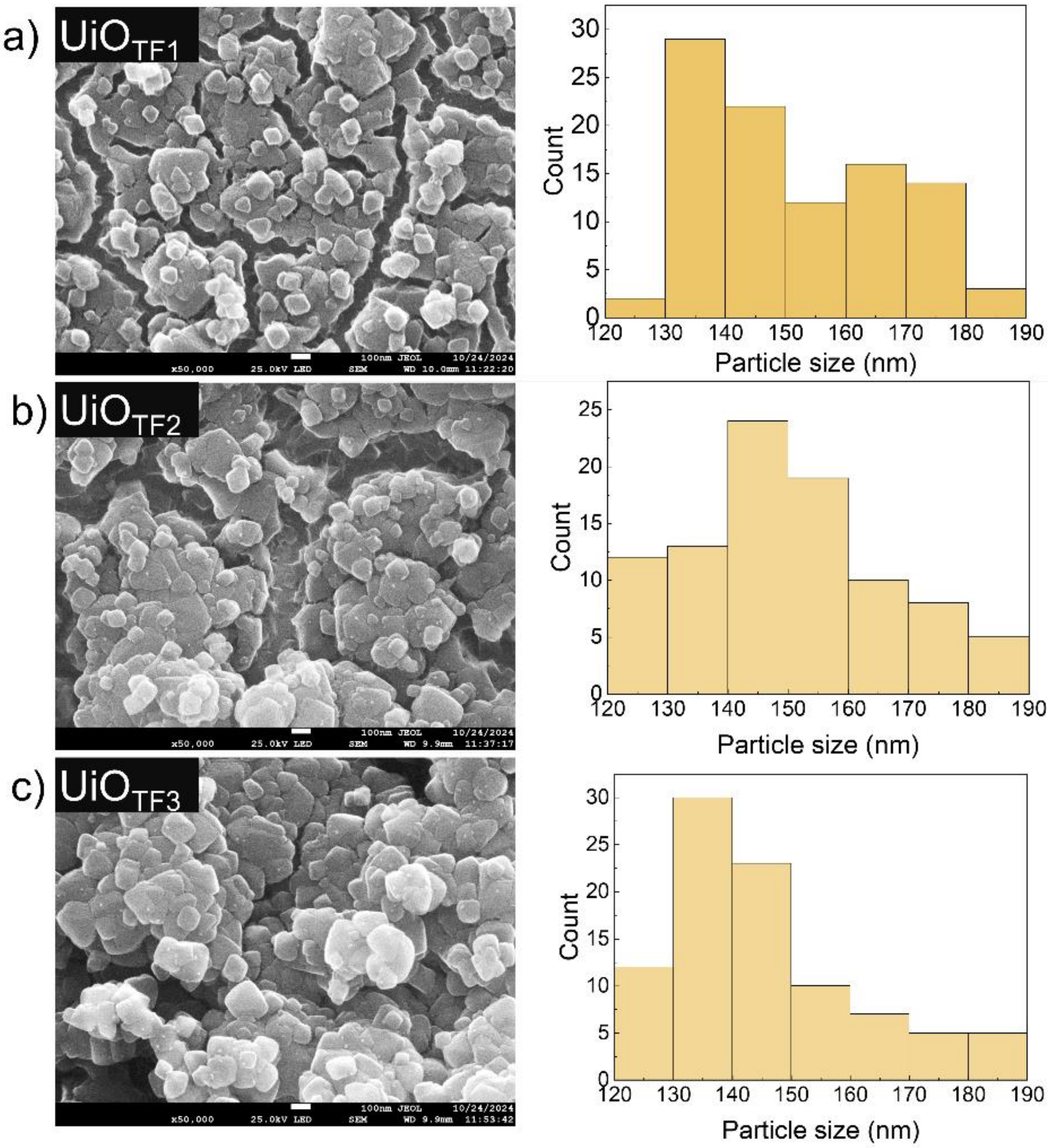

Supplementary Fig. 15. a-c) SEM morphology images and particle size distribution of $UiO_{TF1-3.}$ Note, the cracks are due to beam exposure.

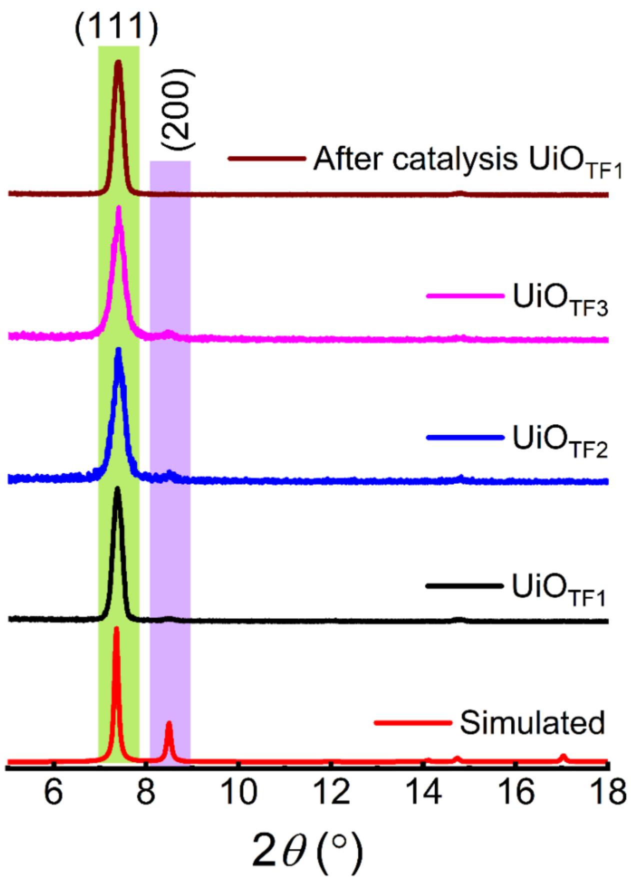

Supplementary Fig. 16. XRD patterns of the different thicknesses of UiO-66-NH_2 thin films.

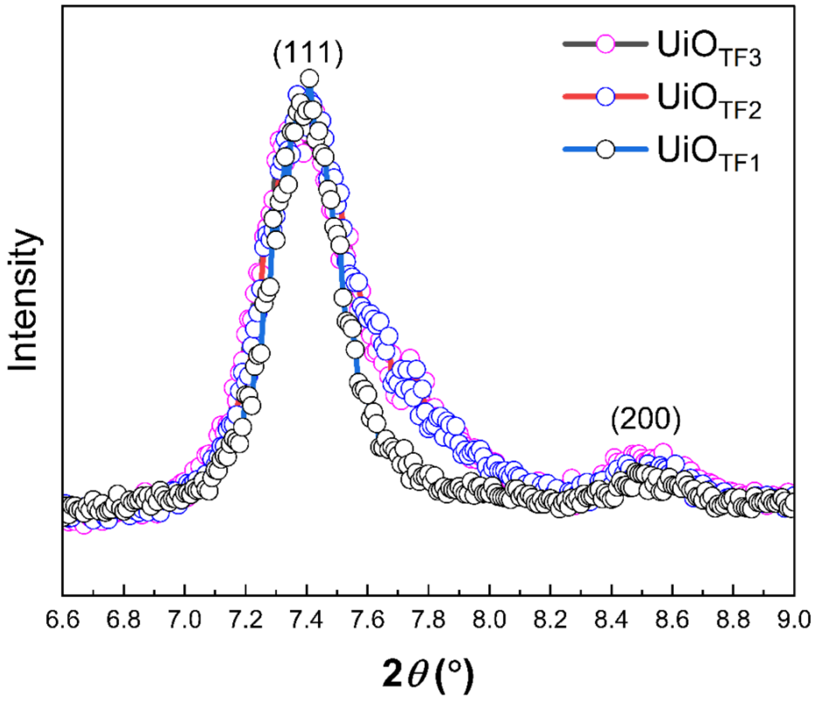

Supplementary Fig. 17. XRD patterns of the UiO$_{TF1-3}$, showing consistent full-width half maximum (FWHM) across all the three samples.

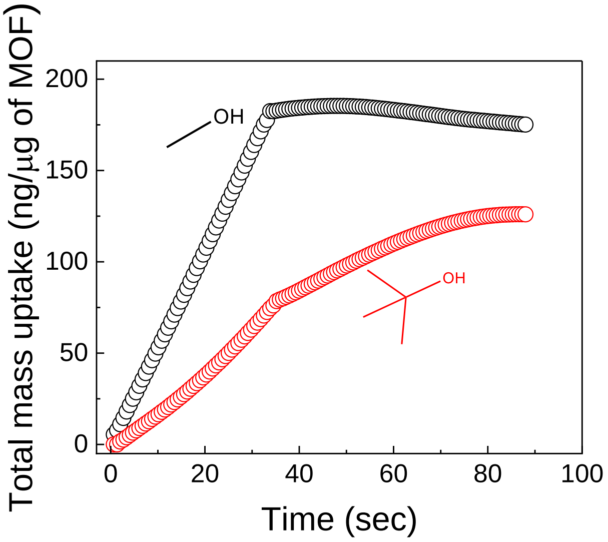

Supplementary Fig. 18. Mass uptake profiles of methanol and *tert*-butanol vapors, for UiO_{TF1} thin film using quartz crystal microbalance experiments at 298 K.

2.9. Cross-flow microfluidic MOF catalysis

2.9.1. Calculation of the catalyst percentage

The percentage of the catalyst loaded in the cross-flow catalytic reactor is calculated from the thickness of the UiO-66-NH_2 thin film and the area of the O-ring.

$$\text{Amount of catalyst} = \pi \times R^2 \times D \times d$$

Where, R= radius of the *O*-ring, D=Thickness of the MOF thin film, d= density of the MOF.

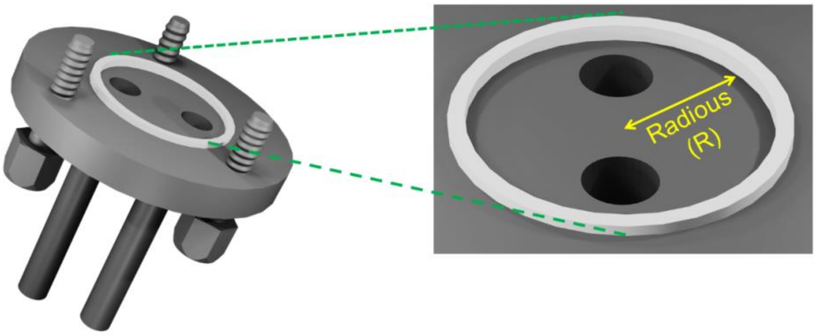

Supplementary Fig. 19. Close view of O-ring in a microfluidic setup.

2.9.2 Catalysis by using UiO_{TF1}, $UiOTF_2$ and UiO_{TF3}

A thin film was placed in the fluidic cell. A solution of 1mmol of benzaldehyde, 1mmol of active methylene compounds, and 10 mL ethanol continuously flowed through the fluidic cell at a flow rate of 5 mL/min. After 24 h, the reaction conversion was monitored using NMR spectroscopy.

Supplementary Table 2. The impact of film thickness on TOF.

**Entry**	**Active methylene compound**	**Thin film (Thickness)**	**Flow rate (mL/min)**	**Amount of catalyst (μg)**	**Yield (%)**	**TOF (h^{-1})**
8	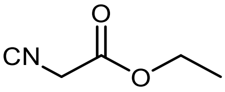	UiO_{TF1} (~120nm)	5	29.5± 2.45	96	2371± 196
9	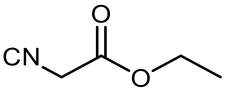	UiO_{TF1} (~120nm)	5	29.5± 2.45	88	2173± 180
10	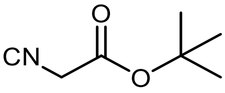	UiO_{TF1} (~120nm)	5	29.5± 2.45	35	864± 71
11	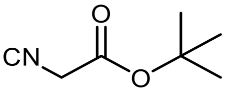	UiO_{TF1} (~120nm)	5	29.5± 2.45	31	765± 63
12	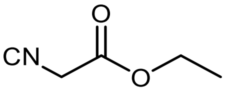	UiO_{TF2} (~300nm)	5	73.9± 7.35	65	642± 127
13	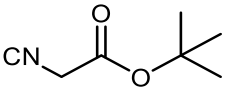	UiO_{TF2} (~300nm)	5	73.9± 7.35	22	217± 22
14	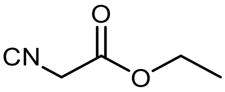	UiO_{TF3} (~400nm)	5	98.5 ± 12.25	66	489± 60
15	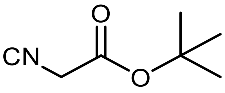	UiO_{TF3} (~400nm)	5	98.5 ± 12.25	15	111± 14

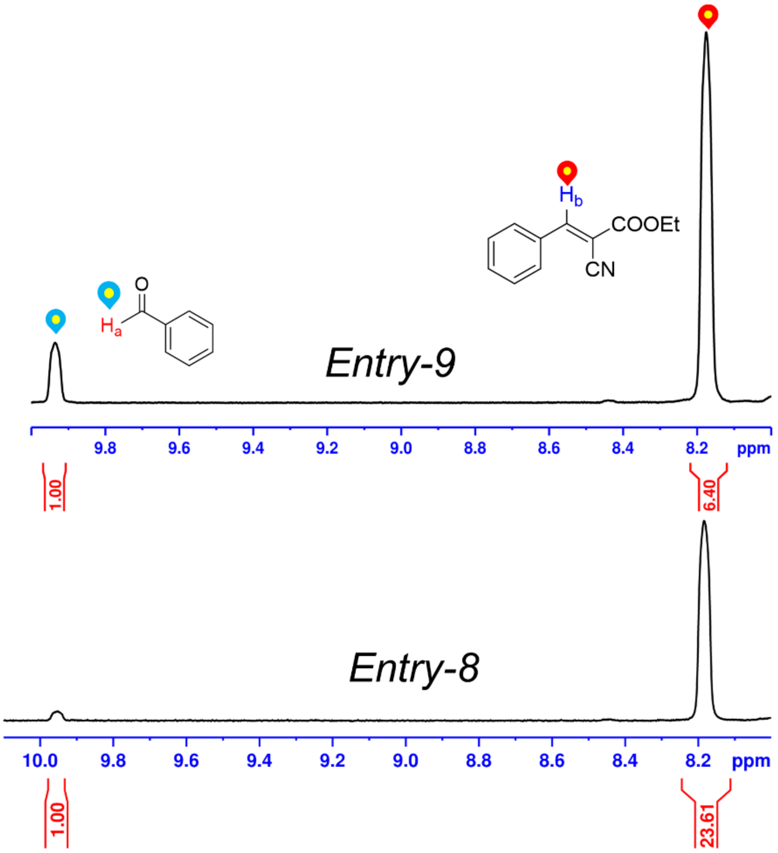

Supplementary Fig. 20. ^{1}H-NMR of Entry 8-9.

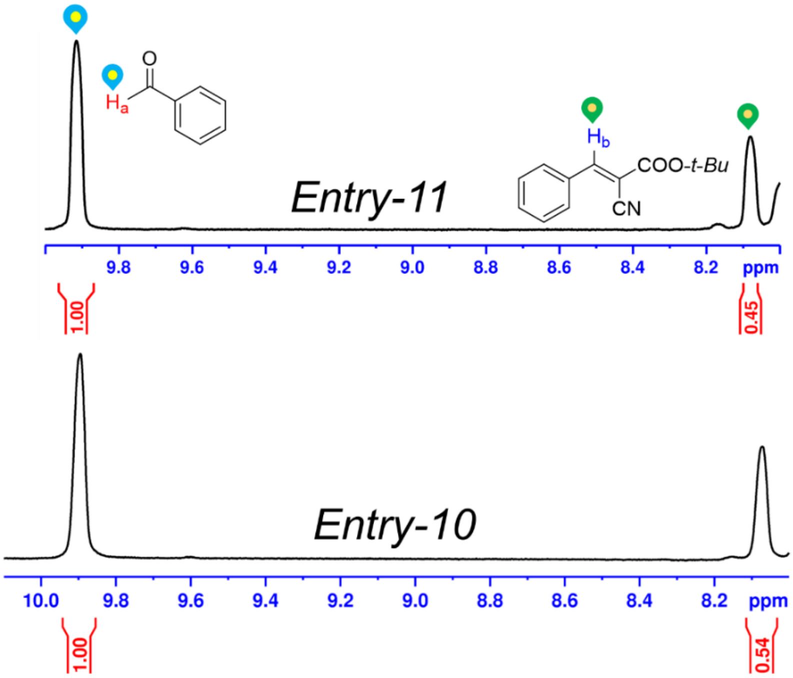

Supplementary Fig. 21. ^{1}H-NMR of Entry 10-11.

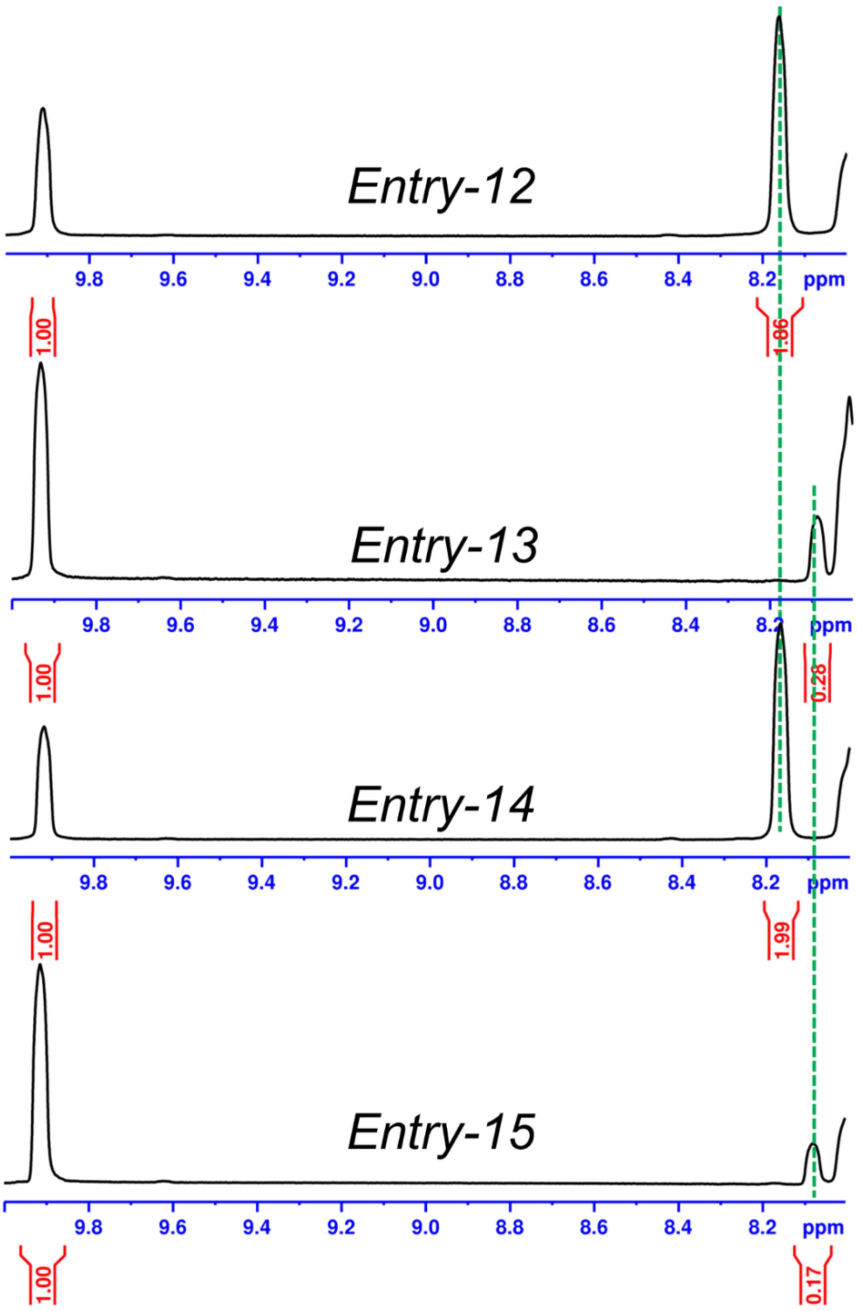

Supplementary Fig. 22. ^{1}H-NMR of Entry 12-15.

2.9.3. One-pot reaction using the catalyst thin film UiO_{TF1}

In a 50 mL test tube, 10 mL of ethanol, 1 mmol of benzaldehyde, and 1 mmol of ethyl 2-cyanoacetate were mixed well. A 1.5×1.5 cm^2 thin film was then dipped into the solution. The reaction was carried out for 4 h at 343 K. The yield of the reaction was subsequently monitored using NMR spectroscopy.

Supplementary Table 3. UiO_{TF1} catalyzed Knoevenagel condensation reaction.

Entry	Thin film	Thickness	Catalyst amount (μg)	Yield (%)
16	UiO_{TF1}	~120 nm	29.5 ± 4.9	50

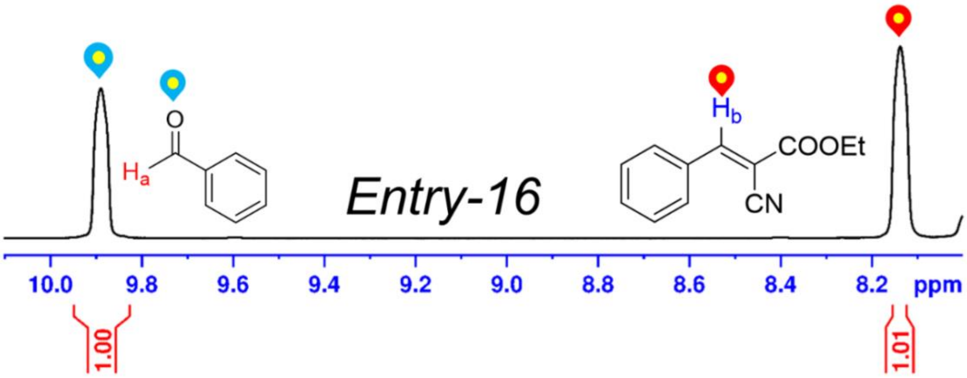

Supplementary Fig. 23. ^{1}H-NMR of Entry 16.

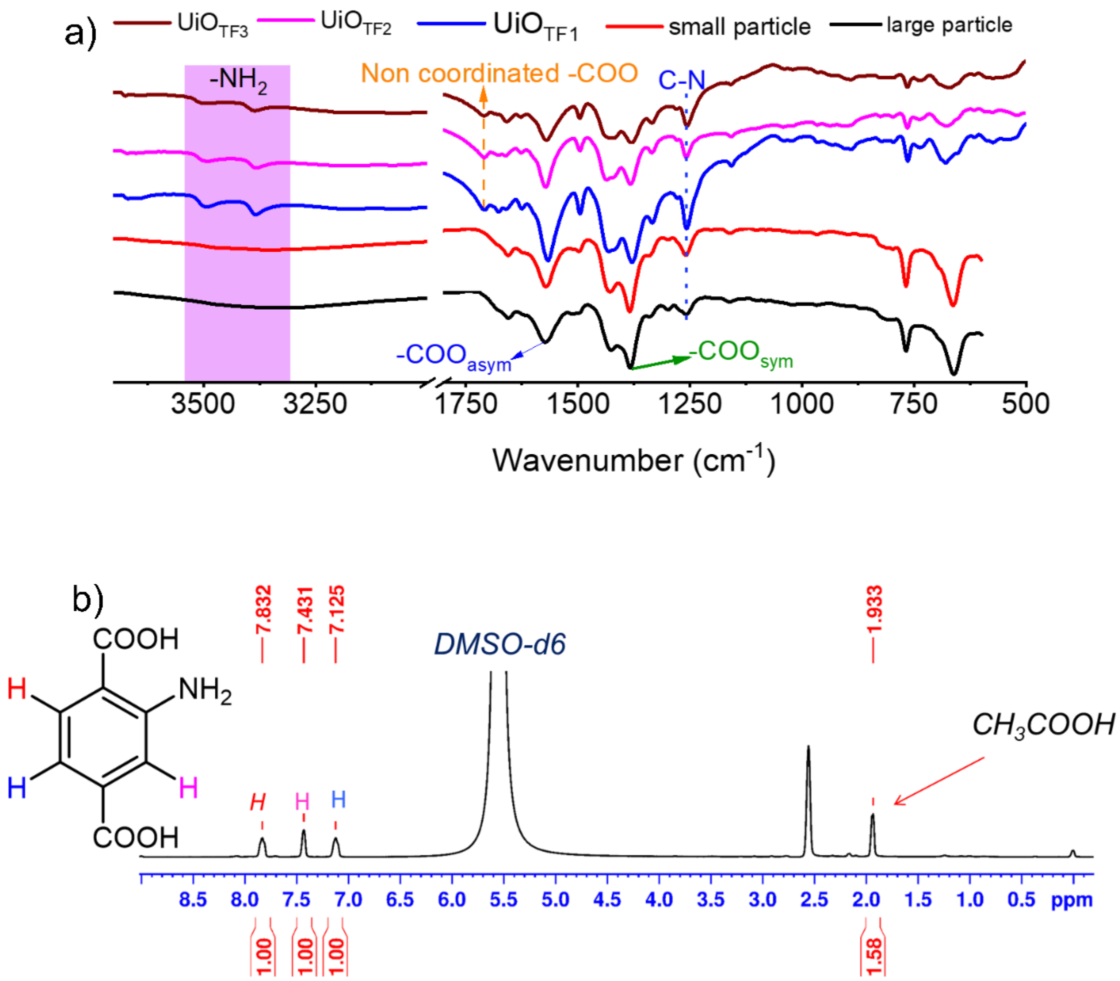

Supplementary Fig. 24. a) ATR and IRRA spectra of the submicron-sized UiO-66-NH_2 crystallites and UiO$_{TF1\text{-}3}$. b) H^1-NMR of UiO-66-NH_2 powder (synthesis condition: 10.77mM metal node, 10.77 mM bdc-NH_2 solution in DMF, RT, 18h) in 50µL HF /0.4ml DMSO-d_6. From the peak area integration, we conclude ~33% missing linker defect (bdc-NH_2:acetic acid ~ 1:0.5). This missing linker defect contributes to a large pore size distribution, shown in supplementary figure 1b.

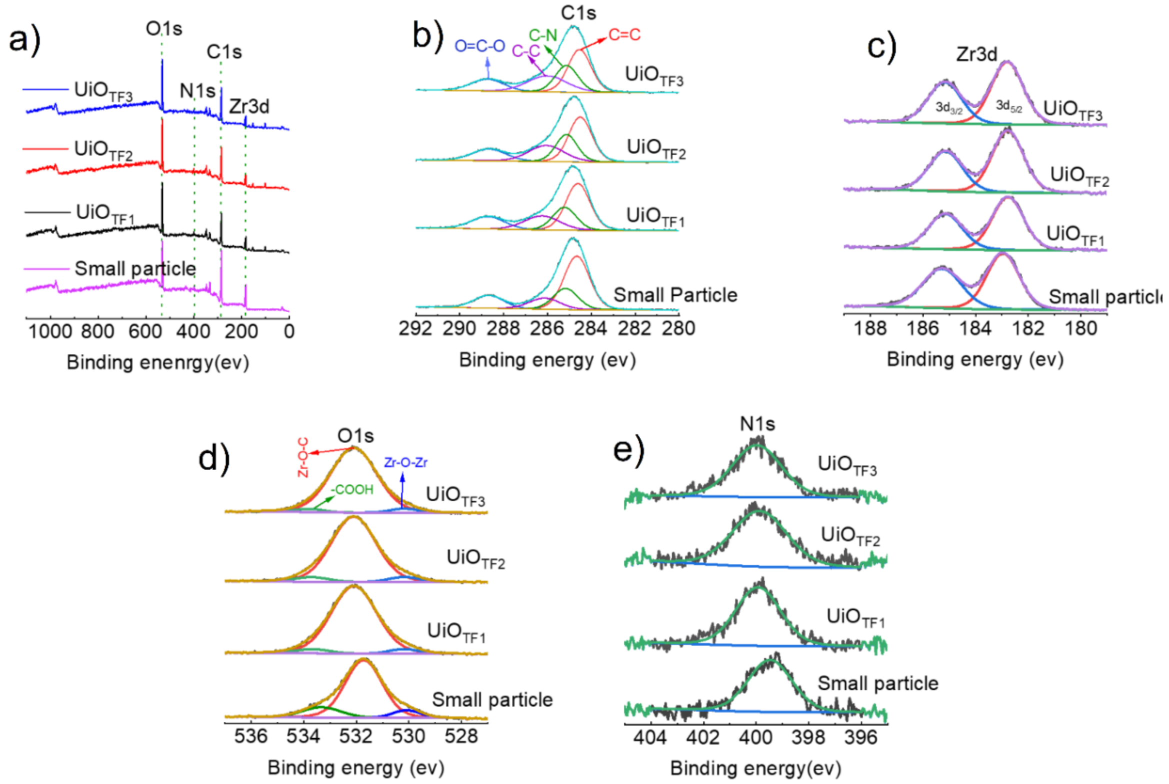

f)

Thin film	Zr-O-C	Zr-O-Zr	-COOH
UiO_{TF1}	1	0.048	0.06
UiO_{TF2}	1	0.041	0.04
UiO_{TF3}	1	0.045	0.04

Supplementary Fig. 25. XPS comparison of the UiO_{TF1-3} and 160 nm crystallites of UiO-66-NH_2. a) overview scan, b-e) high-resolution scan of C, Zr, O, and N respectively. f) area under the peak ratios of Zr-O-C, Zr-O-Zr,-COOH for the thin films.

2.9.4 Flow rate-dependent catalysis using UiO_{TF2}

UiO_{TF2} was placed in the fluidic cell. A solution containing 1mmol of benzaldehyde, 1 mmol of active methylene compound, and 10mL ethanol continuously flowed through the fluidic cell at various flow rates (0.1, 1, 5, 10, 15 mL/min). After 4 hours, the reaction conversions were monitored using NMR spectroscopy.

Supplementary Table 4. Impact of flow rate on yield of the reaction

Entry	Active methylene	Catalyst amount(μg)	Flow rate (mL/min)	Yield (%)	TOF (h^{-1})
17	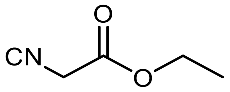	73.9± 7.35	0.1	78	4580
18(a,b,c)	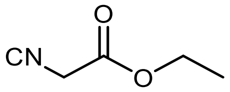	73.9± 7.35	1	72/67/69	4227± 455
19(abc)	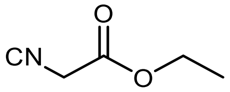	73.9± 7.35	5	45/47/43	2701± 268
20(ab)	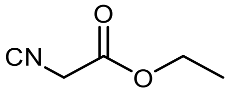	73.9± 7.35	10	35/32	2055± 204
21	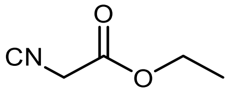	73.9± 7.35	15	31	1820± 181
22	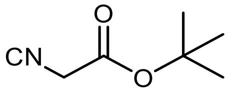	73.9± 7.35	0.1	13	763± 76
23	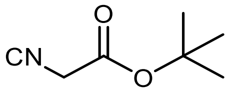	73.9± 7.35	1	6	352± 35
24	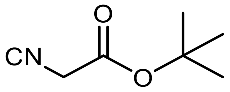	73.9± 7.35	5	5	293± 29

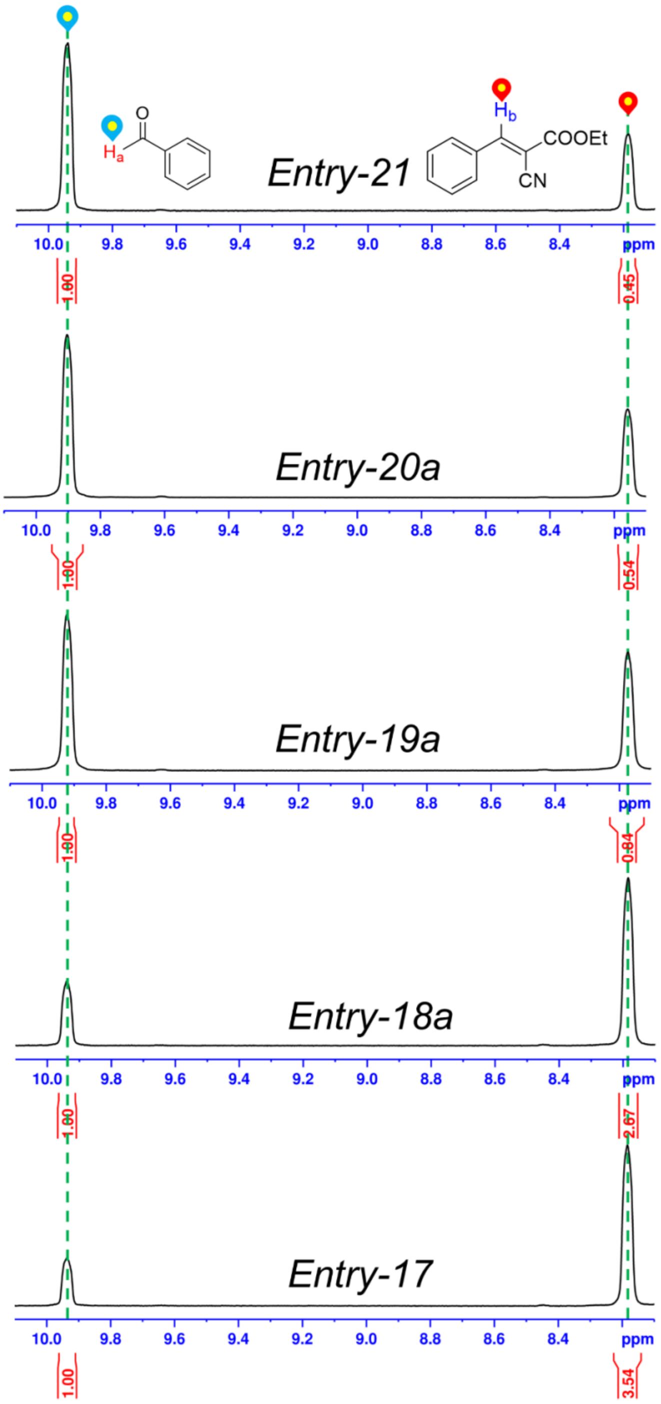

Entry-21
H$_a$
O
H$_b$
COOEt
CN
Entry-20a
Entry-19a
Entry-18a
Entry-17
10.0
9.8
9.6
9.4
9.2
9.0
8.8
8.6
8.4
ppm
1.00
0.45
0.54
0.84
2.67
3.54

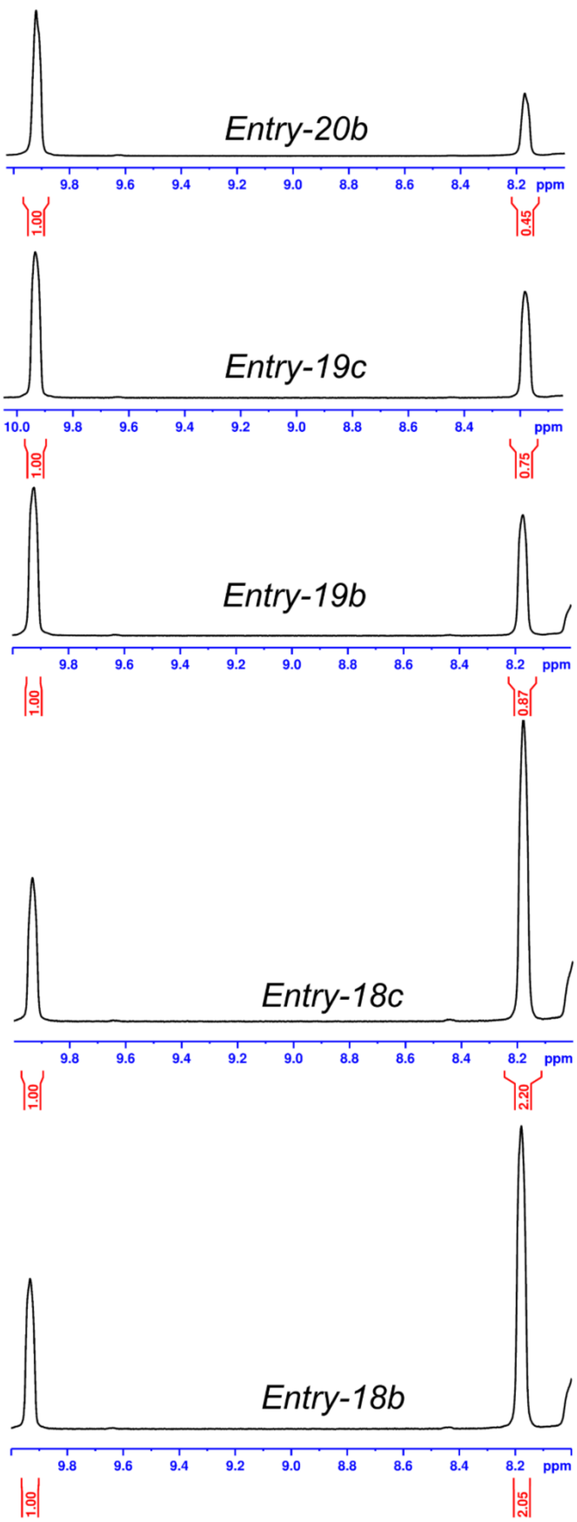

Supplementary Fig. 26. ^{1}H-NMR of Entry 17-21.

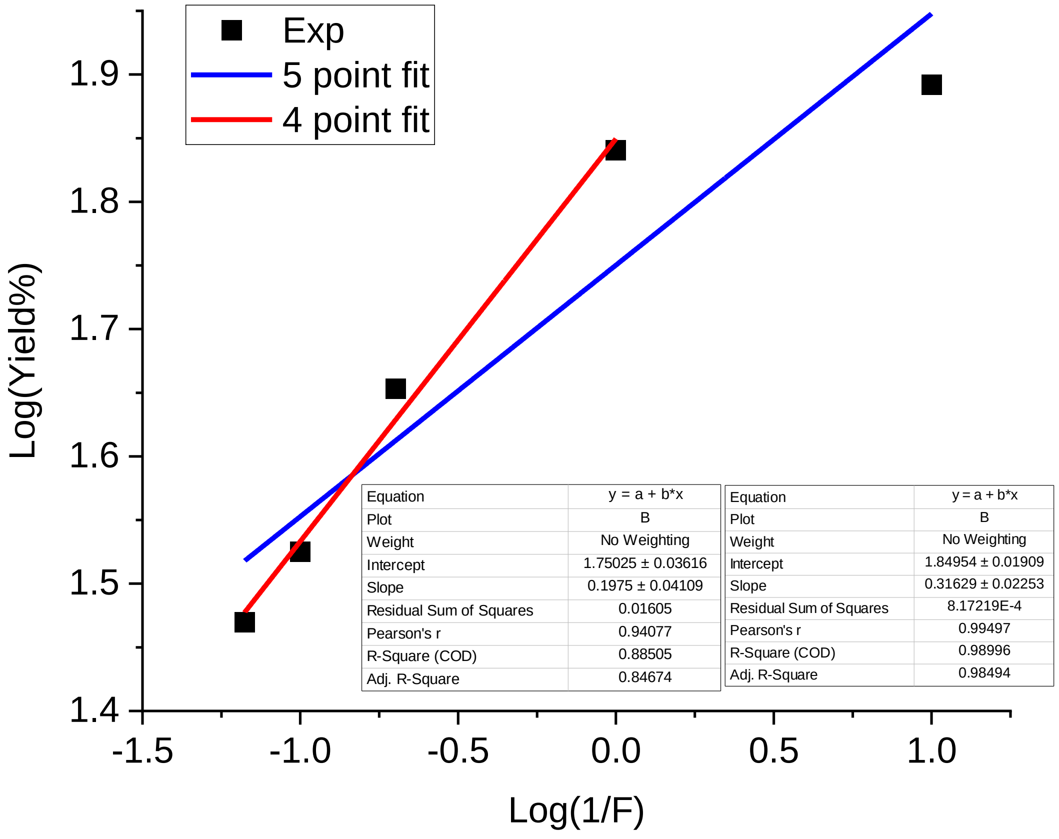

Supplementary Fig. 27. The Log function plot of *Et*-Acr yield % vs 1/F (min/mL) profile (4h reaction at 70 °C). Slope can be in between 0.2-0.32.

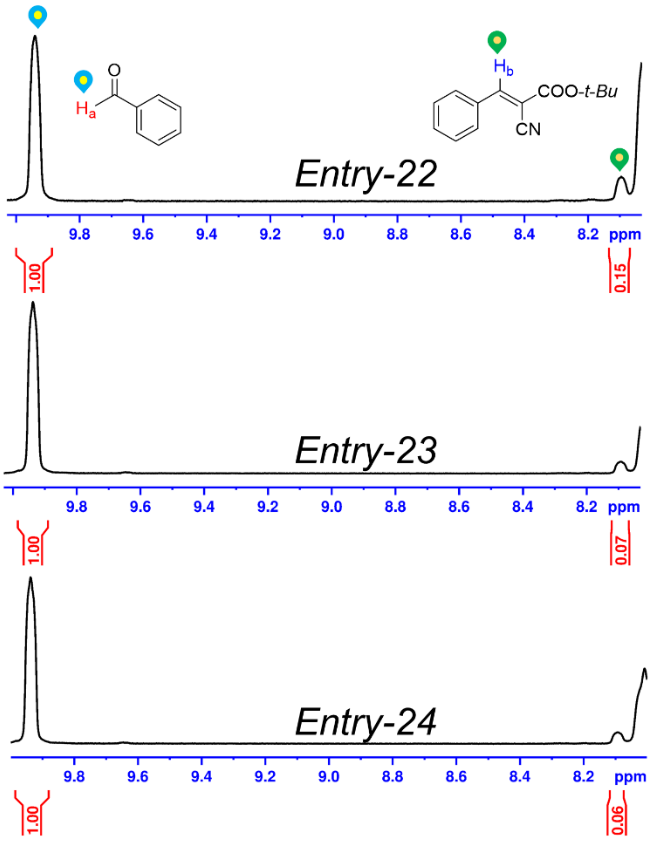

Supplementary Fig. 28. ^{1}H-NMR of Entry 22-24.

Supplementary Table 5. TOF Comparison for 160 nm UiO-66-NH_2 crystallites at 4 h for different active methylene compounds.

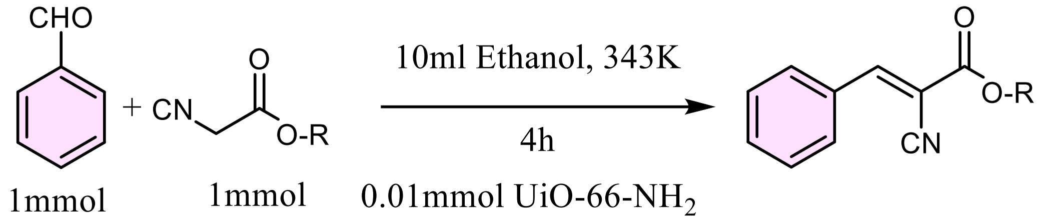

Entry	Active methylene	Crystalite size (nm)	Catalyst amount (mmol)	Yield (%)	TOF (h^{-1})
25	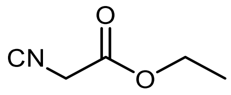	160 ± 50	0.01	19	4.75
26	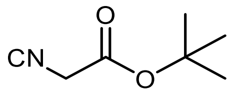	160 ± 50	0.01	4	1

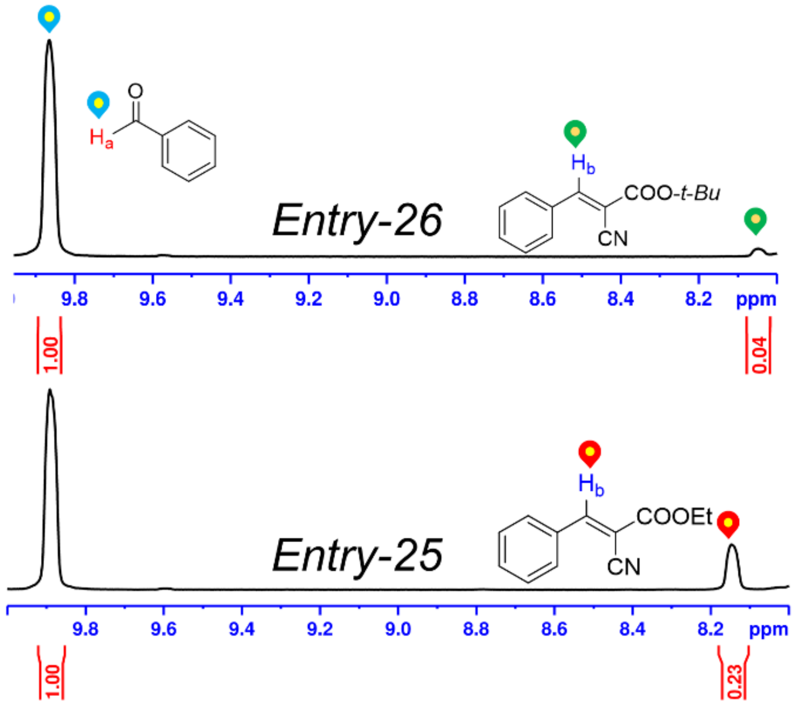

Supplementary Fig. 29. ^{1}H-NMR of Entry 25-26.

Supplementary Table 6. The impact of particle size on the UiO-66-NH_2 catalyzed Knoevenagel condensation for the mixture of substrates (1 mmol benzaldehyde, 0.5 mmol ethyl 2-cyanoacetate, and 0.5mmol *tert*-butyl-2-cyanoacetate).

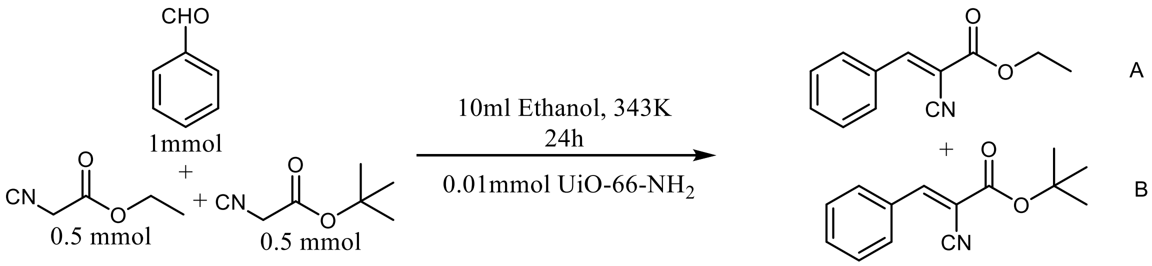

Entry	Particle size of UiO-66-NH_2 powder	A-Yield (%)	B-Yield (%)
27	160 ± 50 nm	55	20
28	500 ± 50 nm	26	6

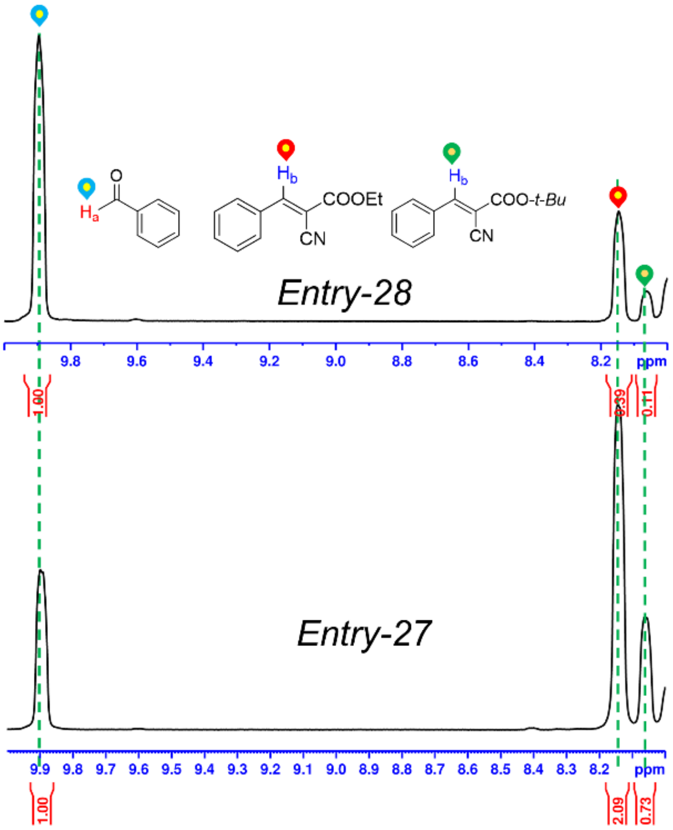

Supplementary Fig. 30. ^{1}H NMR of Entry 27-28.

2.9.5. Catalysis of mixture of substrate using UiO_{TF2}

Supplementary Table 7. The impact thin film on the UiO-66-NH_2 catalyzed Knoevenagel condensation for the mixture of substrates (1mmol benzaldehyde, 0.5 mmol ethyl 2-cyanoacetate, and 0.5 mmol *tert*-butyl-2-cyanoacetate).

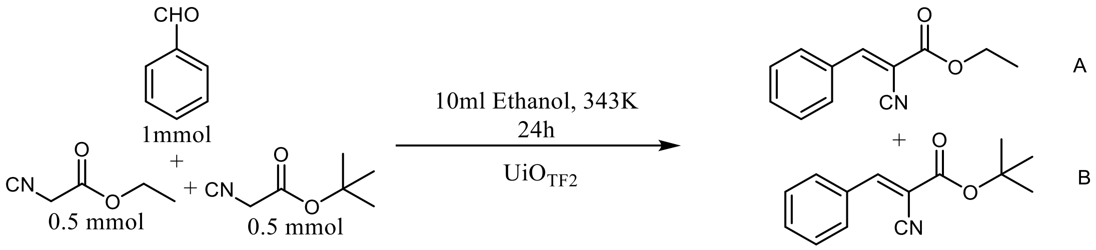

Entry	Thin film(thickness)	Catalyst amount	Flow rate (mL/min)	A-Yield (%)	B-Yield (%)
29	UiO_{TF2}	73.9± 7.35	5	54	10

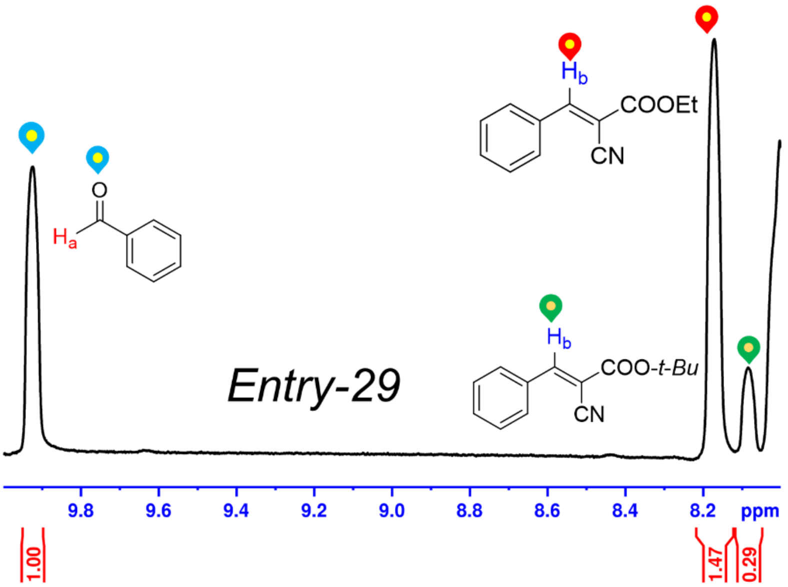

Supplementary Fig. 31. ^{1}H NMR of Entry 29.

Supplementary References:

1 Trickett, C. A. *et al.* Definitive Molecular Level Characterization of Defects in UiO-66 Crystals. *Angewandte Chemie International Edition* **54**, 11162-11167 (2015).

2 Schaate, A. *et al.* Modulated Synthesis of Zr-Based Metal–Organic Frameworks: From Nano to Single Crystals. *Chemistry – A European Journal* **17**, 6643-6651 (2011).

3 Yang, Y., Yao, H.-F., Xi, F.-G. & Gao, E.-Q. Amino-functionalized Zr(IV) metal–organic framework as bifunctional acid–base catalyst for Knoevenagel condensation. *Journal of Molecular Catalysis A: Chemical* **390**, 198-205 (2014).

4 DeStefano, M. R., Islamoglu, T., Garibay, S. J., Hupp, J. T. & Farha, O. K. Room-Temperature Synthesis of UiO-66 and Thermal Modulation of Densities of Defect Sites. *Chemistry of Materials* **29**, 1357-1361 (2017).

5 Panda, S., Kundu, S., Malik, P. & Haldar, R. Leveraging metal node-linker self-assembly to access functional anisotropy of zirconium-based MOF-on-MOF epitaxial heterostructure thin films. *Chemical Science* **15**, 2586-2592 (2024).
